# Supplementary figures and images for: Interleukin-23 levels in umbilical cord blood are associated with neurodevelopmental trajectories in infancy
Source: PLoS One. 2024 Apr 9;19(4):e0301982. doi: 10.1371/journal.pone.0301982 (PMC11003674; doi:10.1371/journal.pone.0301982)

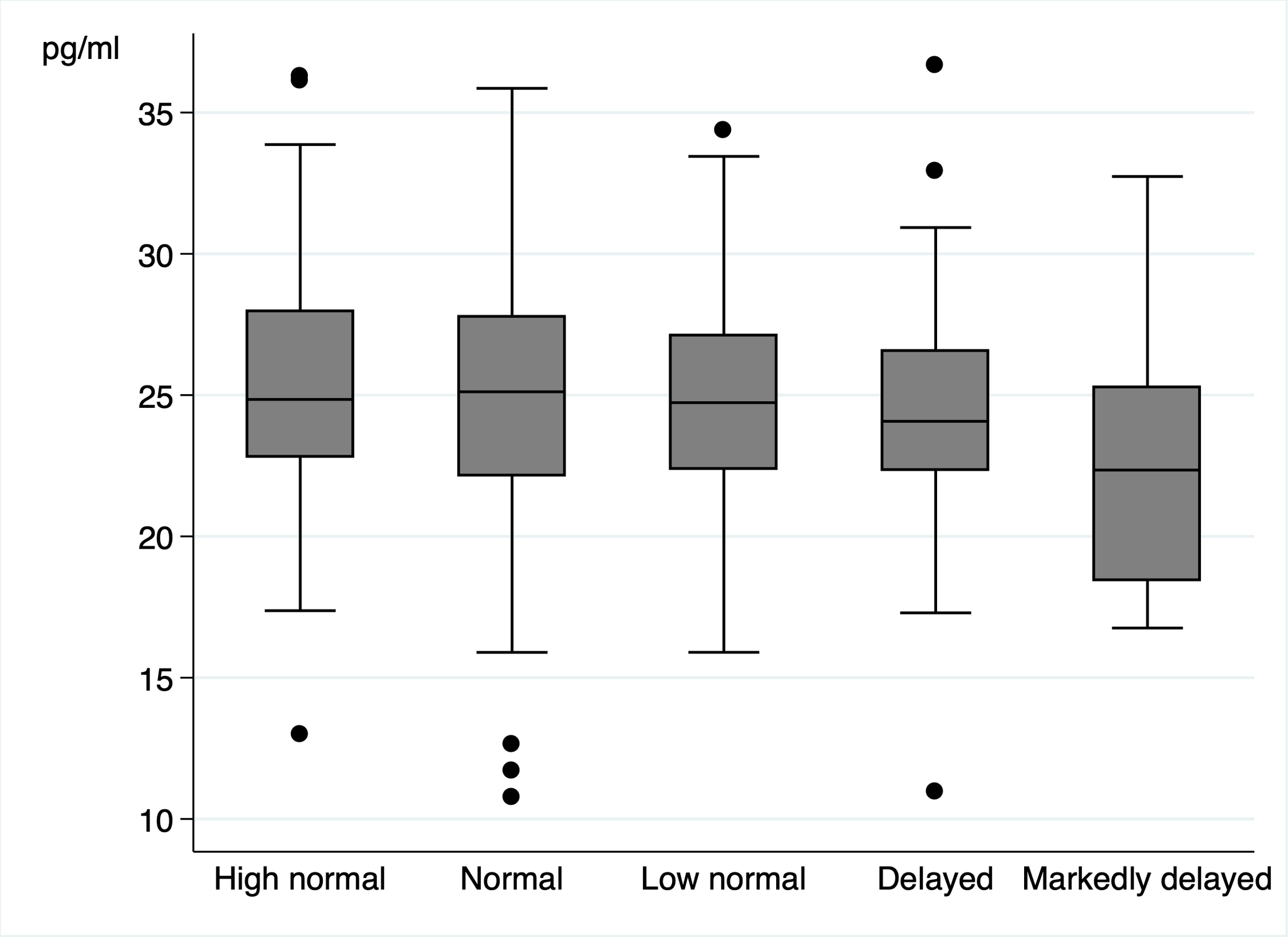

Supplement: S1 Fig — Interleukin-23 concentrations are shown as box-and-whisker plots; midlines indicate medians, boxes indicate interquartile values, whiskers indicate upper and lower adjacent values (the values in the data that are farthest away from the median on either side of the box but are still within a distance of 1.5 times the interquartile range from the nearest end of the box), and dots indicate outside values. (TIF) [file pone.0301982.s001.tif]
